# Basal Cell Carcinoma Infiltrating the Facial Bones—Is It Really a Thing of the Past? Personal Experience over 30 Years and a Review of the Literature

**DOI:** 10.3390/jcm15010254

**Published:** 2025-12-29

**Authors:** Urszula Kozinska, Iwona Chlebicka, Klaudia Knecht-Gurwin, Andrzej Bieniek, Filip Majda, Jacek C. Szepietowski

**Affiliations:** 1 Department of Dermato-Venerology, 4th Military Hospital, 50-981 Wroclaw, Poland; 2 Division of Dermatology, Venereology and Clinical Immunology, Faculty of Medicine, Wroclaw University of Science and Technology, 50-377 Wroclaw, Poland; 3 University Center for General and Oncological Dermatology, Wroclaw Medical University, 50-367 Wroclaw, Poland; 4 Regional Specialist Health Care Center—Diabetology Clinic, Dobrzyńska 21/23, 50-403 Wroclaw, Poland

**Keywords:** basal cell carcinoma, bone invasion, facial tumors, dermatologic surgery, high-risk BCC

## Abstract

**Background/Objectives:** Basal cell carcinoma (BCC) is the most common form of skin cancer, typically exhibiting slow growth and limited metastatic potential. However, in rare, long-standing cases, particularly in high-risk facial regions, deep infiltration into structures such as bone may occur. This study aimed to evaluate whether BCC with bone involvement remains a relevant clinical issue, based on three decades of clinical experience, supplemented by a review of the existing literature. **Methods:** Medical records of patients treated for facial BCC between 1994 and 2025 at a dermatologic surgery department in Lower Silesia were retrospectively reviewed. Among more than 10,000 cases, eight instances of histologically confirmed bone invasion were identified. Clinical and surgical parameters were analyzed, including patient age, tumor size and location, prior treatment and reconstruction method. Relevant literature was incorporated to provide broader clinical context. **Results:** Patients with bone-invasive BCC were elderly (mean age: 75.3 years, SD: 10.94 years) and lesions were typically large (mean diameter 38.9 mm), most frequently located on the nose and forehead. Many cases lacked previous treatment. Smaller nasal tumors were managed with local flaps, while larger lesions on the forehead and temple required skin grafts. Findings from the literature confirm that bone invasion is rare and usually associated with long-standing tumors in anatomically high-risk areas. **Conclusions:** Although rare, BCC with bone infiltration remains a clinically relevant phenomenon, particularly in elderly patients with advanced or recurrent tumors. Early diagnosis, complete excision with histologically clear margins, and individualized surgical planning are essential to prevent deep tissue involvement. Imaging should be reserved for cases in which advanced local invasion is clinically suspected.

## 1. Introduction

Basal cell carcinoma (BCC) is the most frequently diagnosed type of skin cancer and constitutes around 80% of all non-melanoma skin cancers globally [1].

It arises from basal keratinocytes of the epidermis due to cumulative DNA damage and impaired genomic repair mechanisms induced predominantly by ultraviolet (UV) radiation, and is typically characterized by slow, locally invasive growth. BCC very rarely metastasizes; however, in some long-standing cases, it may infiltrate structures located beneath the skin, such as muscles or bone particularly in anatomically complex regions such as the midface [2].

Histological variants of BCC differ significantly in terms of growth dynamics, potential for local invasion and risk of recurrence. The most encountered forms, nodular and superficial BCC, are usually well-demarcated and respond well to standard surgical excision. In contrast, subtypes such as infiltrative, micronodular and morpheaform (sclerosing) BCC are considered high-risk due to their tendency for deeper tissue infiltration and less defined margins [3]. In addition, basosquamous (metatypical) BCC represents a rare variant exhibiting overlapping histopathological features of BCC and SCC (squamous cell carcinoma) and has been associated with more aggressive clinical behavior and an increased potential for deep tissue invasion [3].

Facial BCC, particularly in anatomically complex areas such as the periorbital region, nose and ear has historically been associated with an increased risk of local destruction [1]. In some cases, tumor progression may extend beyond soft tissues to involve deeper structures, including bone. The extent of such involvement appears to be influenced by tumor- and host-related factors. Certain histopathological features including perineural invasion are recognized as markers of aggressive disease and may facilitate tumor spread along nerve pathways, increasing the likelihood of deeper structural involvement including bone [3]. Recognizing the circumstances under which BCC progresses to involve deeper structures is therefore relevant for identifying high-risk patients and guiding appropriate therapeutic decisions.

Although there are no robust epidemiological studies confirming a decrease in the incidence of bone-invasive BCC over time, available literature suggests that such cases are rare and typically associated with prolonged tumor growth and delayed diagnosis [2,4]. Reports of advanced and long-standing BCC with extensive local destruction, including bone involvement are documented predominantly in the form of isolated case reports and small case series [2,4].

In advanced presentations, imaging with computed tomography (CT) or magnetic resonance imaging (MRI) can provide valuable information regarding the extent of BCC in selected cases; however, their routine use in preoperative planning remains controversial [5].

The aim of the review was to compare our long-term clinical observations with data published in the medical literature and to assess whether BCC with bone involvement remains a current clinical problem or rather a rare historical finding.

## 2. Materials and Methods

We retrospectively reviewed medical documentation of all patients treated between 1994 and 2025 with diagnosis of BCC located on the face in a single clinical dermatology and dermatosurgery department in the Lower Silesia region. From this group of more than 10 thousand patients we found eight cases of histologically confirmed BCC infiltrating of facial bones. For each case, the following parameters were analyzed: sex, age at the time of surgery, tumor location, previous treatment, diameter of the lesion, surgical technique used (local flap or skin graft) and year of treatment.

All patients underwent surgical excision of BCC under local anesthesia. When periosteal or bone involvement was identified intraoperatively, the resection was extended to include the affected structures and was managed during the same surgical procedure by an experienced multidisciplinary dermatosurgical and plastic surgical team, without the need for additional consultations with other specialists.

To complement the clinical data and provide a broader perspective, a narrative literature review was conducted. Relevant publications were identified through searches of PubMed, Scopus, Web of Science, and Google Scholar, without restrictions on publication date or article type. The following combinations of keywords were used: basal cell carcinoma, bone invasion, facial bones, deep infiltration and infiltrative subtype. Case reports, case series, and review articles addressing BCC with documented bone involvement were included. Studies with the highest level of evidence and relevance to the discussed topics were selected with the consensus of the authors.

## 3. Results

Between 1994 and 2025, eight patients with histologically confirmed BCC infiltrating the facial bones were identified among more than 10,000 individuals treated for facial BCC at a single dermatologic surgery department. The clinical characteristics of these patients are summarized in Table 1. The mean age at diagnosis was 75.3 years (range: 53–86 years, SD: 10.94 years) and both sexes were equally represented (4 males and 4 females). The majority of tumors were located in high-risk facial areas, with the nose being the most commonly affected site (5 cases), followed by the forehead (2 cases) and the temple (1 case). In most patients (6 out of 8) no prior treatment had been administered, suggesting delayed diagnosis as a significant factor in the progression to bone infiltration. Tumor diameters ranged from 25 mm to 68 mm, with a mean size of 38.9 mm (SD: 16.44 mm). Smaller nasal tumors were managed with local flaps, while larger lesions, particularly those on the forehead and temple, required reconstruction with full-thickness skin grafts. In male patients, nasal localization predominated, whereas females more frequently presented with tumors on the forehead and temple.

Notably, all tumors demonstrated an infiltrative growth pattern on histopathological examination, consistent with their advanced local behavior.

**Figure 1:**
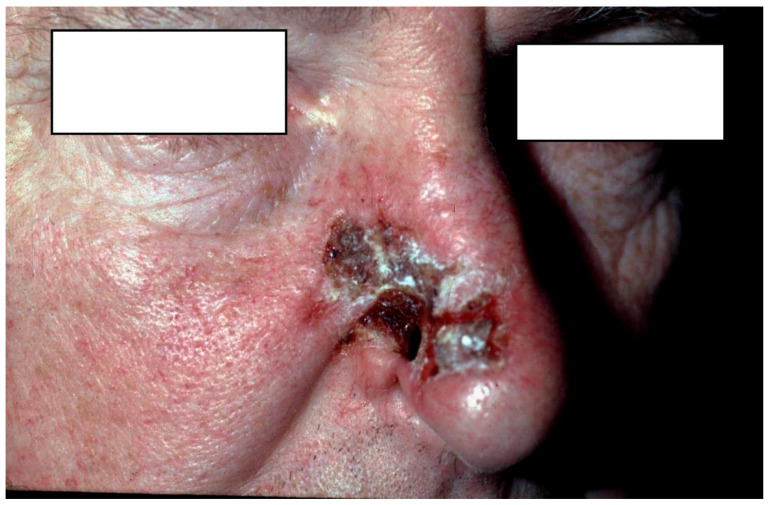
68-year-old male with BCC on the right side of the nasal dorsum and deforming the right ala. The 30-mm lesion was excised under local anesthesia and reconstructed with a local flap.

**Figure 2:**
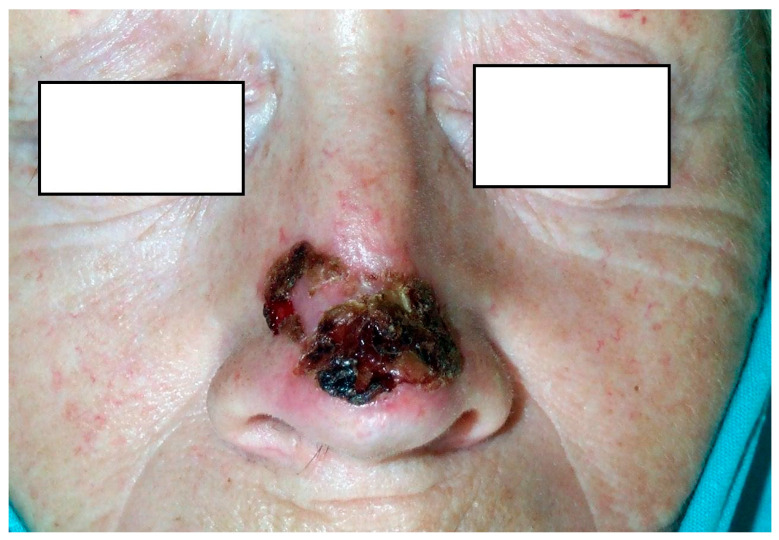
53-year-old female with a 25-mm BCC of the nasal dorsum, extending bilaterally but more extensively on the right side; the lesion was excised under local anesthesia and reconstructed with a local flap.

**Figure 3:**
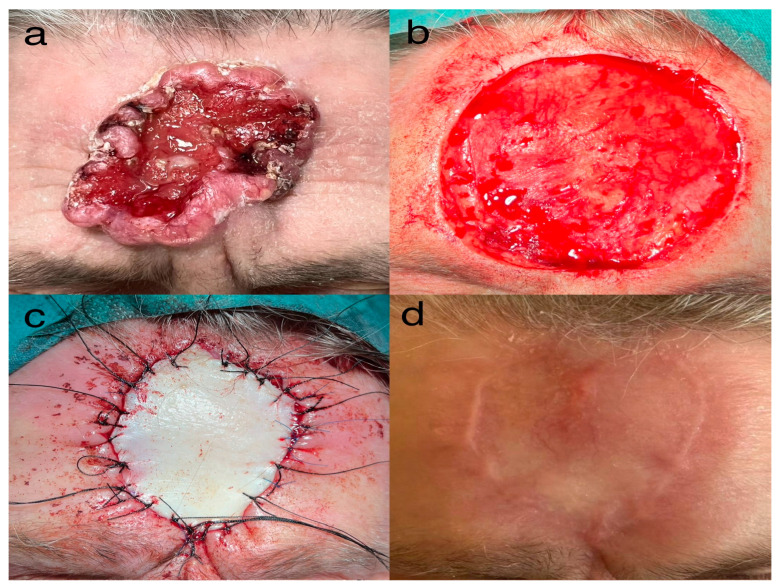
84-year-old female with a recurrent 68-mm BCC of the central forehead; the tumor had developed after incomplete treatment several years earlier and the recurrence remained untreated for a prolonged period. Excision was performed under local anesthesia with full-thickness skin graft reconstruction. (**a**) Lesion prior to surgical excision. (**b**) Intraoperative view following resection. (**c**) Intraoperative view after full-thickness skin graft transplantation from the left supraclavicular region. (**d**) Postoperative outcome at 3-month follow-up. Histopathology in all three cases confirmed the infiltrative/ulcerative subtype, additionally demonstrating periosteal involvement.

Representative cases are shown in Figure 1, Figure 2 and Figure 3.

All patients in this series underwent surgical excision of BCC under local anesthesia. Periosteal or bone infiltration, when present, was identified intraoperatively, as preoperative imaging was not performed in any case. In all instances, the involved bone was thoroughly debrided during the same surgical procedure. The procedures were well tolerated and no significant perioperative complications were observed. Surgical management was adapted to the extent of intraoperative findings in each case. Importantly, histopathological examination confirmed an infiltrative growth pattern in all tumors included in Table 1.

To contextualize these findings, a review of selected case reports and series published between 1986 and 2023 was conducted (Table 2). The literature data revealed a comparable demographic and clinical pattern: patients were generally elderly (typically over 65 years), lesions were most often large (frequently exceeding 30 mm) and located in anatomically complex facial regions such as the nose, orbit, forehead or maxilla. In many published cases, the time from lesion onset to intervention ranged from several years to over two decades, often reflecting delayed presentation or incomplete previous treatment. Advanced surgical approaches including Mohs micrographic surgery, bone resection, flap reconstruction and even orbital exenteration were reported in the management of these deeply infiltrative tumors. Notably, several patients were treated palliatively due to the extent of local destruction or comorbidities.

Together, the institutional experience and literature review support the notion that while bone-invasive BCC is rare, it consistently occurs in the context of long-standing or recurrent tumors situated in high-risk facial zones. These cases often require complex surgical management and carry a considerable risk of morbidity, underlining the importance of early detection and complete excision at the initial stage of disease.

## 4. Discussion

BCC remains the most frequent malignant neoplasm of the skin, yet its clinical behavior can vary widely, ranging from indolent lesions to deeply infiltrative tumors capable of invading cartilage, muscle or even bone. Understanding the factors that contribute to such progression is essential for improving patient outcomes, particularly in older individuals who constitute the majority of those affected. Analysis of our findings within the context of current literature allows a clearer interpretation of risk patterns, diagnostic challenges and therapeutic considerations associated with advanced BCC, particularly in cases demonstrating deep tissue infiltration and bone involvement. Given the clinical consequences associated with delayed diagnosis or incomplete treatment, it is essential to better characterize the clinical, demographic and anatomical factors that may predispose certain patients to more aggressive patterns of tumor growth.

Most of our patients were older (mean age: 75.3 years, SD: 10.94 years), which aligns with the well-established epidemiological pattern of BCC predominating in older adults due to cumulative ultraviolet exposure and age-related immune changes [15].

We found similarities in the literature. Patients with bone-invading BCC were typically older (average age ~65–75 years) with lesions exceeding 2–3 cm in diameter and located in anatomically complex or high-risk areas of the face such as the periorbital region, nasal dorsum and forehead. In several cases the time from onset to diagnosis or definitive treatment ranged from 6 to over 20 years, highlighting the critical role of delayed intervention in enabling aggressive tumor progression.

The nasal region was the most affected site (5 out of 8 cases), which was consistent with literature citing the nose as part of the “H-zone”—a high-risk anatomical area including the nose, periorbital region and nasolabial folds. These regions correspond to embryonic fusion planes, which are characterized by complex anatomy, thinner dermal layers and natural tissue cleavage planes that may facilitate deeper tumor spread and increase the risk of invasion into adjacent structures, including bone [16].

While the sample size is limited, the near-equal distribution of sexes in our cohort supports existing data showing that both men and women are at risk, though the anatomical predilection may vary slightly.

In our treated group men more frequently presented with nasal lesions, whereas women had lesions on the forehead and temple.

Four out of five nasal BCCs occurred in patients without prior treatment, emphasizing the importance of early detection, particularly in these high-risk areas.

Tumor diameters in our group ranged from 25 mm to 68 mm (with an average tumor diameter of 38.88 ± 16.44 mm). A spatial distribution of tumor size was observed, with smaller tumors (25–30 mm) predominantly located on the nose, whereas larger tumors (>50 mm) occurred on the forehead and temple and required skin grafts due to the extent of tissue loss following wide excision. These cases highlight that large tumors, especially in convex or relatively flat anatomical areas, may necessitate more extensive surgical planning, including grafting, to ensure oncologic and aesthetic outcomes.

Surgical treatment methods varied depending on tumor size and location.

Local flaps were preferred in smaller nasal lesions, offering excellent aesthetic outcomes while maintaining oncologic safety. In contrast, larger lesions required skin grafts, particularly in areas with limited tissue laxity (e.g., forehead). This aligns with current surgical standards recommending flap reconstruction in mobile regions and grafts for extensive or poorly vascularized defects.

Notably, in the reviewed cases, over half of the tumors occurred in previously untreated patients, but many others followed incomplete or delayed treatment efforts.

This is consistent with earlier findings by Richmond and Davie, who postulated that residual tumor cells entrapped by scar tissue following incomplete excision may migrate deeper rather than superficially, delaying clinical detection and favoring subclinical progression [17].

Based on our clinical experience over the past 30 years we have encountered a relatively small yet appreciable number of cases. Patients were often elderly, diagnosed late, eventually presenting with deeply invasive BCC infiltrating facial bones.

We observed some differences in clinical presentation, tumor size, recurrence and surgical approach.

All patients in this series underwent surgical excision of BCC under local anesthesia. This approach proved sufficient for tumor removal even in cases where periosteal or bony infiltration was revealed intraoperatively. The procedures were generally well tolerated, with no major complaints of pain during surgery, which emphasizes the applicability of local anesthesia even in more extensive cases. The use of local anesthesia offers a relevant clinical advantage by minimizing perioperative risks and systemic burden, particularly in elderly patients who often present with significant comorbidities.

Preoperative imaging was not performed in any of the patients, as this is not part of the standard of care in Poland and is rarely adopted elsewhere for primary BCC management. Consequently, periosteal or bone infiltration was detected intraoperatively, highlighting the importance of careful direct surgical assessment for establishing the true extent of tumor invasion.

A comprehensive review of the available literature confirms that bone invasion by BCC is a rare but serious clinical occurrence. Most published cases describe locally advanced tumors with deep infiltration in the context of delayed diagnosis or after multiple incomplete excisions.

While BCC is typically considered a slow-growing malignancy with limited metastatic potential, its ability to destructively invade deeper structures, including bone, is well documented, especially in high-risk facial regions such as the orbit, nose and forehead. The specific incidence rate of bone invasion in BCC is not precisely determined in the available literature. However, it is widely recognized as a rare occurrence. For instance, a systematic review and pooled survival analysis by Russell et al. (2022) included 101 patients with BCC exhibiting bone invasion, collected from 70 publications [2]. This study highlights that bone invasion is typically associated with large, neglected tumors located in high-risk facial areas [2]. Interestingly, even after complete surgical resection with negative margins, the authors reported a 30% recurrence rate at five years and disease-specific mortality of 18.2% [2].

Another study by Milenković et al. (2025) analyzed 103 patients with infiltrative BCC of the head and found that 14% exhibited bone invasion [18]. This study identified tumor size, histological subtype, and disease duration as significant factors associated with bone invasion [18]. Tumors with bone involvement were significantly larger in both their longitudinal and transverse tumor dimensions. Specifically, each millimeter increase in tumor length was associated with a 10.2% increase in the odds of bone invasion (OR = 1.102, *p* < 0.001).

Additionally, infiltrative and morpheaform histological subtypes were more likely to necessitate surgical bone removal [18]. Surgical management varied depending on tumor extent and location. Many patients required extensive resections involving bone, followed by reconstruction with local flaps, skin grafts or free tissue transfer [18]. In certain cases, especially with orbital or intracranial invasion, enucleation was necessary.

In line with these findings, tumors in the present series showed a heterogeneous size distribution depending on anatomical location with smaller lesions predominantly observed on the nose and larger tumors more frequently affecting the forehead and temple.

Several studies have suggested that incomplete excision of BCC may not only increase the risk of recurrence but could also promote more aggressive tumor behavior.

Richmond and Davie proposed that fibrotic changes induced by scarring may impair the ability of residual malignant cells to migrate superficially, favoring instead deeper tumor progression. This phenomenon can delay clinical detection of recurrence and complicate subsequent surgical management, particularly in anatomically sensitive facial areas [17].

While these studies provide valuable insights, the exact incidence rate of bone invasion in BCC across all cases remains undetermined. Despite significant advances in diagnostic and therapeutic approaches, the persistence of such advanced cases underscores the importance of early detection and treatment to prevent potential complications associated with deep tissue invasion.

Our clinical observations align with these findings. We have encountered cases where delayed diagnosis and treatment allowed BCCs to progress to stages involving bone invasion. These instances often required complex surgical interventions, including bone resection and reconstructive procedures, underscoring the importance of early detection and management.

Importantly, all tumors in our series were located along embryonic fusion sites of the face, a finding that needs particular emphasis. These regions represent anatomical junctions formed during facial embryogenesis, where multiple tissue planes converge and persist as pathways of reduced resistance in adult anatomy. Tumor growth along these fusion planes has been suggested as a potential mechanism facilitating subclinical extension and deeper infiltration of BCC even when superficial tumor dimensions appear limited. The fact that all bone-invading BCCs in our cohort arose within embryonic fusion sites suggests that tumor location itself may play an important role in determining aggressive local behavior. This finding is consistent with previous reports identifying high-risk facial zones as preferential locations for locally destructive tumor growth [16].

BCC remains a clinically relevant neoplasm not only because of its high incidence, but also due to the substantial local morbidity associated with delayed diagnosis and inadequate initial management. The continued occurrence of advanced BCCs observed both in our practice and in the available literature further emphasizes the need to strengthen patients’ education on consistent photoprotection and the importance of seeking prompt dermatologic evaluation for any atypical or evolving skin lesions as these measures remain essential for minimizing avoidable tumor progression and reducing the likelihood of extensive surgical management. Moreover, the presence of bone-invasion tumors demonstrates that, despite being classified as a locally malignant neoplasm, BCC must be taken seriously as its potential for deep local invasion should not be underestimated and should be clearly communicated not only to patients but also to healthcare professionals as part of comprehensive preventive and clinical education. Clinicians performing dermatologic surgery should remain vigilant to the possibility of underlying bone infiltration as its detection may necessitate a notably broader surgical approach and more extensive excision than initially anticipated.

Comparing our clinical experience with published cases further allows us to identify consistent trends in tumor behavior and therapeutic strategies that tend to be preferred. Such comparisons help to illustrate how deeply infiltrative BCC manifests in day-to-day practice, particularly when extending to osseous structures. This broader view improves our diagnostic awareness and supports more tailored treatment planning.

### 4.1. The Role of Imaging in Assessing Bone Infiltration

In cases of suspected deep invasion, especially when BCC is located in high-risk areas such as the nose, orbit or forehead, imaging may be considered helpful to clinical and histopathologic assessment. Although not routinely used for most cases, radiologic evaluation is particularly valuable when bone involvement or perineural spread is suspected based on clinical findings.

Computed tomography (CT) with bone window settings is considered the most effective modality for detecting erosion or destruction of the underlying bone structures, while magnetic resonance imaging (MRI) is preferred for assessing soft tissue extension and possible perineural infiltration [4]. However, CT may be helpful in identifying obvious bone destruction, but its sensitivity for detecting early or limited cortical invasion is relatively low. Wang et al. report that histologically confirmed osseous invasion can occasionally occur without corresponding radiographic findings, particularly in early or subtle cases. Conversely, imaging features such as sclerotic changes or bone remodeling do not always correlate with tumor infiltration and may instead reflect chronic pressure or age-related anatomical changes [5].

These observations suggest that while CT and MRI are useful in selected cases, especially when deep invasion is suspected, it is not recommended in the routine preoperative evaluation of BCC. Their use should be limited to advanced, recurrent cases where clinical suspicion of deep or complex infiltration is high [19].

One such high-risk site is the orbit. Orbital invasion by periocular BCC, though uncommon, is associated with significant ocular morbidity and, in rare cases, may lead to life-threatening complications. Importantly, orbital invasion can remain clinically silent, especially in patients with recurrent or previously incompletely excised tumors [11]. In these situations, CT and MRI play a key role in identifying subtle or subclinical bone and soft tissue involvement. However, in most cases, particularly when dealing with well-demarcated, small tumors, clinical examination and histopathological analysis remain sufficient for establishing appropriate surgical plans.

### 4.2. Systemic and Adjuvant Treatment Options

Although surgery remains the most commonly used treatment for BCC, other therapeutic options such as radiotherapy and targeted therapy with vismodegib, a selective hedgehog pathway inhibitor, are also available. In selected cases, particularly those with positive or narrow surgical margins, perineural invasion, or other high-risk histopathological features, adjuvant radiotherapy may be considered to reduce the risk of local recurrence [20]. While vismodegib represents a valuable systemic treatment option, its use remains restricted within current therapeutic programs to patients with locally advanced, inoperable or metastatic BCC [21]. In addition to vismodegib, sonidegib represents another hedgehog pathway inhibitor approved in the European Union for the treatment of locally advanced BCC [22]. However, despite its registration, it is not currently reimbursed in Poland. For patients who do not respond to or cannot tolerate hedgehog pathway inhibitors, the anti-PD-1 monoclonal antibody cemiplimab is available as a second-line systemic therapy and can also be assessed in Poland [23]. In our case series, despite the presence of bone invasion, all tumors were deemed resectable and were managed surgically without the need for systemic therapy.

An increasing body of evidence highlights the need for careful multidisciplinary evaluation when selecting the optimal treatment strategy for BCC, particularly in anatomically challenging locations or in tumors demonstrating aggressive patterns of growth. In practice, these decisions must take into account not only tumor stage and histopathological characteristics but also patient comorbidities, aesthetic outcomes and the need to preserve function. For locally advanced lesions, systemic therapies have significantly expanded the therapeutic landscape, offering meaningful responses in cases previously considered unsuitable for surgery or radiotherapy. Nevertheless, these agents are associated with a considerable toxicity profile, including muscle spasms, dysgeusia, alopecia and fatigue [24]. In addition, their high cost along with limited reimbursement further challenge their practical integration into routine clinical care. Consequently, surgery remains the cornerstone of management whenever possible, as it provides high rates of local control with acceptable morbidity. The outcomes observed in our cohort support this principle, demonstrating that even in the presence of deeper structural invasion complete surgical excision can remain both achievable and effective.

## 5. Conclusions

BCC of the face, although commonly regarded as a low-risk malignancy, can occasionally present with aggressive features, including deep infiltration and, in rare instances, bone involvement. Our retrospective case series highlights the persistent clinical relevance of advanced or recurrent BCCs, particularly in elderly patients and in anatomically high-risk regions such as the nose and forehead. While the incidence of bone-invasive BCC remains low, these cases often require extensive surgical intervention and long-term follow-up. BCCs with bone invasion are often large, long-standing tumors that tend to recur despite treatment efforts. A careful clinical assessment supported by histopathology remains the gold standard in routine management, while imaging techniques should be reserved for situations with a strong clinical suspicion of deep tissue invasion.

Based on our 30 years of clinical experience and available literature, we emphasize the importance of early diagnosis, complete excision with histologically clear margins and individualized treatment planning. Furthermore, the persistence of advanced cases, including those progressing to deep tissue and bone involvement, reinforces the need for ongoing education of both patients and healthcare professionals regarding diligent photoprotection and the early evaluation of atypical or evolving skin lesions, as these measures remain critical in preventing avoidable tumor progression. Continued awareness of the potential for aggressive behavior in certain BCC subtypes remains essential for optimizing both functional and oncologic outcomes.

## Figures and Tables

**Table 1:** Summary of Patients treated between 1994–2025 in the Department of Dermatosurgery with BCC with Facial Bone Invasion. The anatomical site of BCC is provided together with the corresponding facial bone involved, indicated in parentheses.

No. of Case	Sex	Age	Site of BCC	Previous Treatment	Diameter of BCC [mm]	Type of Surgical Therapy for BCC	Year of Treatment
1 (Figure 1)	M	68	Nose (nasal bone)	no	30	Local flap	1994
2	M	76	Nose (nasal bone)	yes	30	Local flap	1994
3	M	74	Nose (nasal bone)	no	25	Local flap	1996
4	M	76	Nose (nasal bone)	no	28	Local flap	1998
5	K	86	Temple (temporal bone)	no	55	Skin graft	1998
6 (Figure 2)	K	53	Nose (nasal bone)	no	25	Local flap	2001
7	K	85	Forehead (frontal bone)	no	50	Skin graft	2007
8 (Figure 3)	K	84	Forehead (frontal bone)	yes	68	Skin graft	2025

**Table 2:** Summary of Published Cases of BCC with Bone Invasion. Table summarizing selected case reports and series of basal cell carcinoma (BCC) with confirmed bone invasion, based on literature indexed in PubMed between 1986 and 2023. Data includes patient demographics, lesion location, treatment history, tumor size, surgical approach, and time from lesion appearance to intervention. NR—not reported in the source publication; the exact year of treatment could not be determined.

No.		Sex	Age	Site of BCC	Previous Treatment	Diameter of BCC [mm]	Type of Surgical Therapy for BCC	Year of Treatment	Time to Intervention
1	Mikhail et al., 1986 [6]	F	78	forehead/scalp	Yes	large	Mohs, flaps	1986	-
2	Mikhail et al., 1986 [6]	M	54	temple/zygomatic/orbit	-	large	Mohs	1986	-
3	Mikhail et al., 1986 [6]	F	60	orbit/ethmoid	Yes	-	Mohs, grafts	1986	~20 years
4	Ko et al., 1992 [7]	M	61	right face/dura	No	170 × 120	None (palliative)	1992	6 years
5	Ko et al., 1992 [7]	F	70	scalp/forehead/nose	No	200 × 150	Radiotherapy + excision + grafts	1992	7 years
6	Ko et al., 1992 [7]	F	82	glabella/nose/eyelids	No	large	Graft + radiotherapy	1992	15 years
7	Nagler and Laufer, 2001 [8]	None	None	mandible/maxilla (3 cases)	None	None	mandibular/maxilla resection	NR	None
8	Aygit et al., 2006 [9]	M	61	oral commissure/mandible	Yes	30 × 30	Mandibular resection + flap	NR	~10 years
9	Aygit et al., 2006 [9]	M	65	orbit/maxilla	Yes	20 × 20	Bone graft + free flap	NR	~3 years
10	Aygit et al., 2006 [9]	F	70	nasal dorsum	No	15 × 15	Flaps	NR	~20 years
11	Aygit et al., 2006 [9]	M	65	maxilla	No	25 × 10	Implant + flap	NR	~10 years
12	Aygit et al., 2006 [9]	F	67	orbit/zygomatic	No	60 × 60	Palliative	NR	None
13	Mathieu and Fortin, 2005 [10]	F	66	forehead	Yes	80 × 80	frontal one resection flap, cranioplasty	2005	5 years
14	Leibovitch et al., 2005 [11]	None	70 ± 13	orbit (64 cases)	84% recurrent	-	Orbital exenteration	2005	3.5–7.8 years
15	Kleydman et al., 2009 [12]	F	87	nose → calvarium	Yes	7 × 5	Flap	2008	7 years
16	Kleydman et al., 2009 [12]	F	87	nose → cranial nerves	Yes	7 × 5	Flap + RT	2009	6 years
17	Mozaffary et al., 2015 [13]	F	50	chin/mandible	No	90 × 55	Flap	2015	20 years
18	Russell et al., 2022 [2]	None	None	(101 cases)	-	-	-	2022	-
19	Bengoa-González et al., 2024 [14]	None	None	orbit (7 cases)	84% recurrent	-	Exenteration ± vismodegib	2011–2023	-

## Data Availability

All data supporting the findings of this study are included in the article. Additional patient data are not publicly available due to ethical and privacy restrictions but may be provided by the corresponding author upon reasonable request.
